# Role of Conformational Dynamics of Sulfotransferases SULT1A1 and SULT1A3 in Substrate Specificity

**DOI:** 10.3390/ijms242316900

**Published:** 2023-11-29

**Authors:** Daniel Toth, Balint Dudas, Maria A. Miteva, Erika Balog

**Affiliations:** 1 CiTCoM UMR 8038 CNRS, INSERM U1268 MCTR, Université Paris Cité, 75006 Paris, France; toth.daniel@phd.semmelweis.hu (D.T.); b.dudas@ucl.ac.uk (B.D.); 2 Department of Biophysics and Radiation Biology, Semmelweis University, 1094 Budapest, Hungary; 3 Department of Physics and Astronomy, University College London, London WC1E 6BT, UK

**Keywords:** sulfotransferase, SULTs selectivity, molecular dynamics, docking, drug–drug interactions

## Abstract

Sulfotransferases (SULTs) are phase II metabolizing enzymes catalyzing the sulfoconjugation from the co-factor 3′-Phosphoadenosine 5′-Phosphosulfate (PAPS) to a wide variety of endogenous compounds, drugs and natural products. Although SULT1A1 and SULT1A3 share 93% identity, SULT1A1, the most abundant SULT isoform in humans, exhibits a broad substrate range with specificity for small phenolic compounds, while SULT1A3 displays a high affinity toward monoamine neurotransmitters like dopamine. To elucidate the factors determining the substrate specificity of the SULT1 isoenzymes, we studied the dynamic behavior and structural specificities of SULT1A1 and SULT1A3 by using molecular dynamics (MD) simulations and ensemble docking of common and specific substrates of the two isoforms. Our results demonstrated that while SULT1A1 exhibits a relatively rigid structure by showing lower conformational flexibility except for the lip (loop L1), the loop L2 and the cap (L3) of SULT1A3 are extremely flexible. We identified protein residues strongly involved in the recognition of different substrates for the two isoforms. Our analyses indicated that being more specific and highly flexible, the structure of SULT1A3 has particularities in the binding site, which are crucial for its substrate selectivity.

## 1. Introduction

Human cytosolic sulfotransferases (SULTs) are a family of enzymes of phase II metabolism catalyzing the conjugation [1] of the sulfuryl moiety from their co-factor, 3′-Phosphoadenosine 5′-Phosphosulfate (PAPS), to hydroxyl or primary amino group of substrates [2,3,4]. SULTs metabolize a large diversity of small molecules including endogenous compounds such as neurotransmitters and hormones, natural compounds [5], environmental toxins and drugs [3,4] helping protect the body from toxic effects or drug–drug interactions. SULTs, like other major drug metabolizing enzymes [6,7] exhibiting a broad range of substrates, show important structural plasticity, which has been extensively studied in recent decades [2,8,9,10]. SULTs include 13 enzymes classified into five families in humans, SULT1, SULT2, SULT3, SULT4 and SULT6, which are present in different tissues [11]. A number of polymorphisms have been identified for SULTs that may be critical for inter-individual variability in drug response and toxicity or for increasing disease risk [12], and it has been shown that some of them critically affect the flexibility and the dynamics of the protein [13].

Previous studies have determined that SULT1A1 is the most abundant SULT isoform [3,8,14] predominantly concentrated in the liver but also expressed in the lungs, platelets, kidney, and gastrointestinal tissues. It exhibits a broad substrate range with specificity for small phenolic compounds including drugs such as acetaminophen and minoxidil, and pro-carcinogens such as N-hydroxyl-aromatic and heterocyclic aryl amines [3,14]. SULT1A3 is expressed in the highest concentration in the intestine, but it is also found in the brain, the lung, and the platelets and in a very low concentration in the liver [15]. SULT1E1, also known as estrogen sulfotransferase, has a unique role in hormone homeostasis, as it sulfonates both estrogens and iodothyronines [11]. We calculated 50% sequence identity of SULT1E1 with SULT1A1, and 58% sequence identity of their binding pockets. Similarly, SULT1E1 shares 49% sequence identity with SULT1A3, and their binding site share 52% sequence identity. These differences explain the higher specificity of SULT1E1 compared to SULT1A1 and SULT1A3. SULT1A1 and SULT1A3 share 93% sequence identity of their whole structures, and 73% sequence identity of their binding pockets. Despite their similarities, SULT1A1 shows a wider substrate range than SULT1A3. SULT1A3 displays a high affinity toward monoamine neurotransmitters like dopamine or the cardiac drug, isoprenaline [16,17]. Therefore, elucidating the factors that determine the substrate specificity of the SULT1A1 and SULT1A3 isoenzymes is crucial for understanding the molecular mechanisms guiding their selectivity, and for better predicting their substrates and inhibitors.

**Figure 1:**
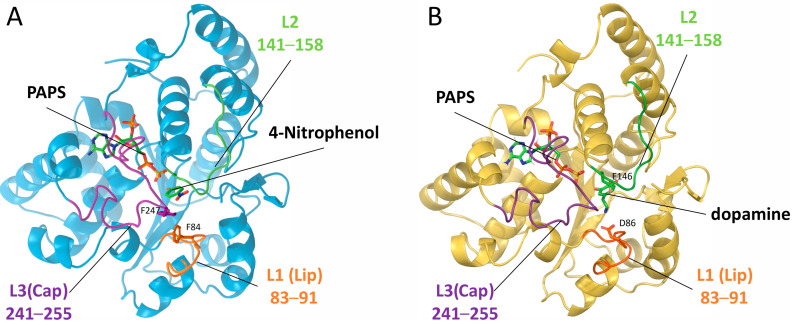
Crystal structures of human sulfotransferases. (**A**) SULT1A1 (in sky blue, PDB ID: 4GRA). The representative ligand 4-nitrophenol not present in 4GRA is inserted for illustration. (**B**) SULT1A3 (in gold, PDB ID: 2A3R). The active co-factor PAPS, and the representative ligands 4-nitrophenol and dopamine are shown in sticks. For both structures, the co-crystalized inactive co-factor PAP was replaced by PAPS. The three loops gating the active sites are indicated L1(Lip) in orange, L2 in green, and L3(Cap) in magenta. Residues important for substrate recognition are labelled and represented by sticks.

The active sites of the SULT1 isoenzymes comprise three loops of significant importance, namely L1 (also known as the Lip), the opposite to it; L2, both being important in the recognition and binding of substrates; and the largest loop L3 (also referred to as the Cap), strongly involved in the co-factor binding [18] (see Figure 1). The loops’ fluctuations, previously identified to participate in the gating mechanism [18,19,20], play a key role in the ligand accommodation and orientation [21,22].

In order to elucidate the molecular mechanisms guiding SULT1 selectivity, in this work, we have studied the dynamic behavior and structural specificities of SULT1A1 and SULT1A3, utilizing molecular dynamics (MD) and MDeNM (molecular dynamics with excited normal modes) simulations, as well as ensemble docking of a large number of experimentally validated ligands. Our results have shown that the two isoenzymes exhibit surprisingly different dynamical behavior, in particular at the loops L2 and L3, and have indicated amino acid residues of the binding pocket strongly involved in the ligand interactions and specificity.

## 2. Results and Discussion

### 2.1. Structural Analysis of SULT1A1 and SULT1A3

Exploring the dynamic behavior of the two isoenzymes is essential to understand SULT1 specificities; therefore, we conducted an in-depth analysis using all-atom MD simulations. We studied three systems: (I) SULT1A1+PAPS; (II) SULT1A3+PAPS; and (III) SULT1A3+PAPS+dopamine. The simulations for SULT1A1+PAPS were executed in our previous work [23], wherein we have performed three 200ns long MD simulations with different initial velocity distributions. Here, we present additional analyses on those SULT1A1 simulations together with analyses on the SULT1A3 systems in order to compare the dynamics of SULT1A1 and SULT1A3. In our previous work, we observed that the MD simulations showed the relatively rigid behavior of SULT1A1, and that MD was insufficient to explore the conformational changes allowing the proper accommodation of large substrates such as the drug fulvestrant; therefore, a more efficient method, molecular dynamics with excited normal modes (MDeNM) [24], was additionally used. MDeNM involves several simultaneous MD simulations in which different randomized linear combinations of the most relevant low-frequency normal modes (gained via all-atomic normal mode analysis) are kinetically activated and therefore allowed to pass energy barriers more efficiently. This method achieved a better exploration of the conformational space of SULT1A1.

Here, we performed three replicas of 500ns long MD simulations for SULT1A3+PAPS that were sufficient to extensively explore the conformational space (see below for details). Furthermore, to elucidate the substrate binding, an additional three replicas of 500 ns long MD simulations with the bound ligand dopamine (SULT1A3+PAPS+dopamine) were also performed. The root mean square deviation (RMSD) values calculated for the backbone-heavy atoms are presented in the Supplementary Materials (Figure S1) for all studied systems, and show the overall rigidity of SULT1A1 during the MD simulations, and the high flexibility of SULT1A3 during the first 200 ns and the 500 ns of the simulations, in the presence or absence of a bound substrate.

**Figure 2:**
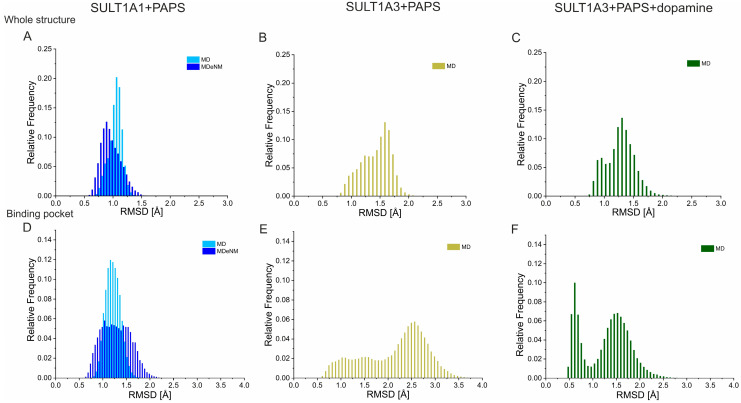
The root mean square deviations (RMSD) of MD simulations for SULT1A1 and SULT1A3 with respect to the corresponding crystal structures. RMSD are calculated on the backbone of the whole protein (**A**–**C**) and on the binding pocket (residues listed in Supplementary Materials) heavy atoms (**D**,**E**). For SULT1A1+PAPS (**A**,**D**), light blue represents the MD, while dark blue represents the MDeNM-generated data. (**B**,**E**) represent MD simulations of SULT1A3+PAPS. (**C**,**F**) represent MD simulations of SULT1A3+PAPS+dopamine.

In order to compare the conformational space explored by SULT1A1 and SULT1A3 during the performed simulations, in Figure 2, we represent the relative frequency of the RMSD values of the two isoforms. Figure 2A,D show the MD and MDeNM simulations of SULT1A1+PAPS. The presented MD data (light blue) indicate a population distribution that can be described by a single, relatively narrow peak centered at 1.12 Å in the case of the whole protein structure (Figure 2A), and 1.22 Å for the binding pocket (Figure 2D) (binding pocket residues are listed in the Supplementary Materials). This suggests a constrained exploration of the conformational space around a single energy minimum. Conformations mapped by the MDeNM simulation (dark blue) are more dispersed, varying between 0.6 Å and 1.5 Å for the whole protein, and between 0.6 Å to 2.3 Å for the binding pocket, thus indicating a more exhaustive conformational sampling. This behavior suggests that the conformations are distributed around different local energy minima, indicating multiple subpopulations. Indeed, while a single Gaussian could be fitted to the relative frequency of RMSD for the MD data, multiple-Gaussian fitting was necessary for the MDeNM data (Figure S2A,D).

It is obvious that the MD-generated conformational sampling of SULT1A3 in the presence or absence of a bound substrate is much wider compared to SULT1A1. The RMSD values calculated for the whole protein of SULT1A3 without dopamine vary between 0.8 Å and 2.0 Å (Figure 2B), and between 0.6 Å and 3.5 Å for the binding pocket (Figure 2E), which is even larger than the MDeNM conformational mapping of SULT1A1. The Gaussian fitting of the histogram for SULT1A3+PAPS suggests three distinct subpopulations (Figure S2B,E). These results clearly indicate the higher conformational flexibility of SULT1A3 compared to SULT1A1. The MD simulations of the dopamine-bond SULT1A3 structure show a range of RMSD values for the whole protein (Figure 2C), similar to those for the substrate-free SULT1A3; however, the RMSD of the binding pocket is reduced in the presence of the bound dopamine, varying between 0.4 Å and 2.5 Å (Figure 2F). The Gaussian fitting proposed that the substrate binding restricted the conformational space onto two populations (Figure S2C,F). These data confirmed that the MD simulations performed for SULT1A3 achieved sufficiently exhaustive conformational sampling.

**Figure 3:**
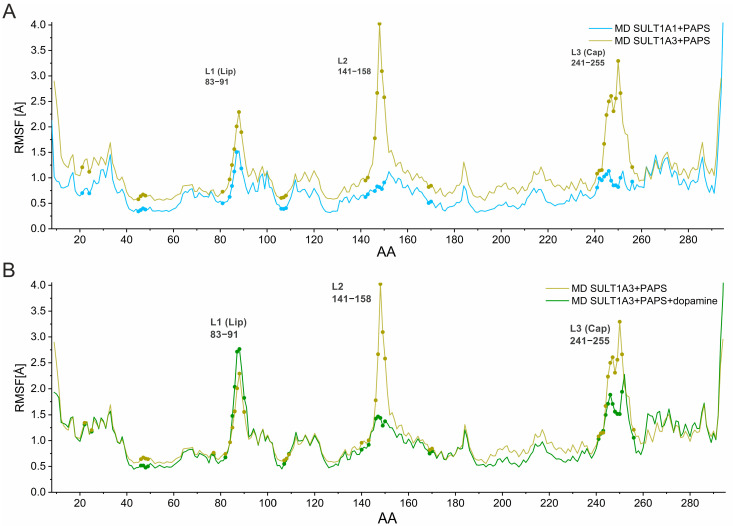
Root mean square fluctuations (RMSF) of the Cα atoms of the amino acid residues (AA) for the MD simulations performed on SULT1A1 and SULT1A3. (**A**). RMSF of SULT1A1+PAPS (blue) and SULT1A3+PAPS (gold). (**B**). RMSF of SULT1A3 without (gold) and with bound dopamine (green). Residues of the binding pocket are indicated with dots.

To identify and characterize the structural elements responsible for the RMSD variations described above, we represent the root mean square fluctuation (RMSF) of the Cα atoms for the MD simulations performed for both isoforms in Figure 3.

Figure 3A compares SULT1A1+PAPS (blue) and SULT1A3+PAPS (gold). The functional loops L2, L3 of SULT1A3 exhibit significantly higher fluctuations than those of SULT1A1. As a comparison, the residue A148 (in loop L2) of SULT1A3 shows an RMSF value of 4.0 Å, while the corresponding residue V148 of SULT1A1 shows a very low RMSF value of 0.9 Å; the residue H250 of L3 loop (SULT1A3) shows an RMSF value of 3.3 Å, whereas I252 (SULT1A1) exhibits RMSF of 1.1 Å.

Figure 3B shows the RMSF of the MD simulations for the two SULT1A3+PAPS systems, with (green) and without (gold) dopamine. The fluctuations of L1 seem to be marginally effected by the presence of dopamine. It is particularly interesting that the presence of dopamine led to a significant reduction of the L2 RMSF decreasing from 4.0 Å to 1.4 Å for A148. The RMSF also decreased for L3. These findings clearly indicate the stabilizing effect of dopamine on the L2 and L3 loops. Our findings of the large flexibility of L3 of SULT1A3 corroborate previously reported results [18,25]. In fact, experimentally, it was shown that the absence of the PAPS in crystal structures of SULT1A3 leads to a disordered L3 [8,25].

**Figure 4:**
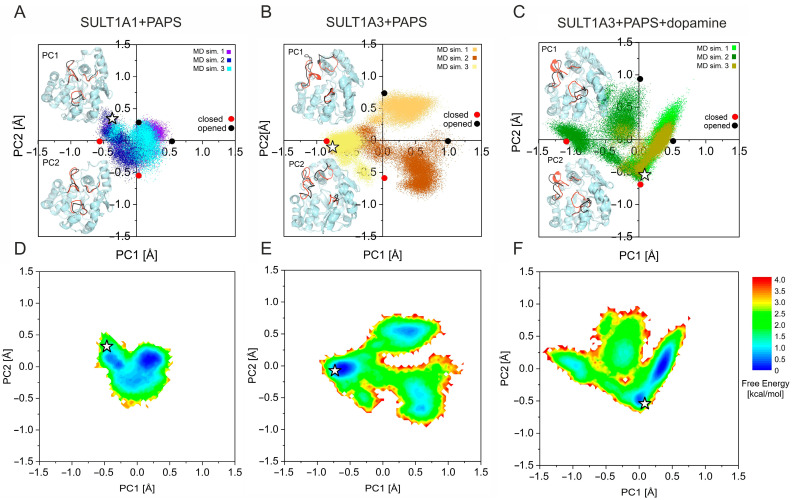
PCA analysis. Population distributions of conformers generated by MD of SULT1A1+PAPS (**A**), SULT1A3+PAPS (**B**) and SULT1A3+PAPS+dopamine (**C**), projected onto the subspace covered by PC1 and PC2. In the insets, structures corresponding to the extremities of PC1 and PC2 are represented, and their location on the diagram is indicated by circles. The functionally important loops L1, L2, and L3 are color-coded: open-loop conformations are depicted in black, while closed ones are depicted in red. Free energy landscapes (FELs) of SULT1A1+PAPS (**D**), SULT1A3+PAPS (**E**), and SULT1A3+PAPS+dopamine (**F**) are in the space defined by PC1 and PC2. The initial structures are denoted by white stars.

In order to have a more detailed view of the conformational space mapped by the three systems, we also performed principal component analysis (PCA) calculations. After calculating the principal components (PCs) of the simulation trajectories, we projected the conformers of the simulations onto the subspace covered by the first two PCs (ranked by the corresponding variances), i.e., PC1 and PC2. Figure 4 shows the results of this projection for the three systems. Directions of PC1 and PC2 are represented so that more positive values correspond to more open conformations.

PCA plots show very constrained fluctuation of the conformations along both PC1 and PC2 for SULT1A1 (between −0.5 and 0.5 Å), and a much wider conformational sampling for both SULT1A3 systems. In the absence of bound dopamine, SULT1A3 has a 4.5 times higher variance than SULT1A1 (see Table S1 of Supplementary Materials) for both along PC1 and PC2. These results are consistent with the RMSD results (Figure 2).

For both ligand-free and dopamine-bound SULT1A3, the projections of the conformations onto PCs vary in the same magnitude (from −1 to 1 Å along PC1, and from −1 to 0.75 Å along PC2 for SULT1A3+PAPS; from −1.25 to 0.5 along PC1, and from −0.75 to 1 along PC 2 for SULT1A3+PAPS+dopamine). However, the variance along both PC1 and PC2 is considerably higher for the dopamine-free system, further emphasizing the stabilizing effect of the presence of dopamine in the binding pocket. Furthermore, the projections of the dopamine-bound SULT1A3 conformations are more directional, also suggesting more constrained dynamics, as discussed above on the RMSD of the binding pocket in the presence of dopamine.

By plotting the 3D structure of the conformations corresponding to the extremities of PC1 and PC2, it can be seen that for the three systems, the first two PCs are mainly localized to the functionally important loops L1, L2, and L3, and the directionality of the PCs correspond to the opening and closing of the loops. For SULT1A1+PAPS, the significant opening/closing of L1 can be detected, in agreement with the RMSF results seen in Figure 3. For SULT1A3+PAPS, the three loops exhibit opening/closing movements, L2 in particular shows a large amplitude motion, which contributes to the large conformational sampling, also in agreement with the RMSF results. The binding of dopamine stabilizes the L2 in its closed conformation, and loops L1 and L3 are still flexible, in agreement with the RMSF results.

We have also transformed the distribution of conformations discussed above in the free energy landscapes (FEL) representations, which are shown in Figure 4D–F. While the FEL of SULT1A1 could be described by one shallow energy minimum, i.e., many energy minima positioned closed to each other being interconnected with low energy barriers, the energy landscape of both SULT1A3 forms show distinct energy minima being separated by relatively higher energy barriers. The energy barriers separating the energy minima are higher for the dopamine-bound SULT1A3 than for the ligand-free form, which is also a sign of the constraint on the loop movements resulting from the dopamine binding.

The above discussed results clearly demonstrate that SULT1A1’s dynamics are more restricted compared to those of SULT1A3, particularly in the loops L2 and L3; one can speculate that this may play a role in the selectivity mechanism. This could be interpreted as the relative rigidity of SULT1A1 helping to retain diverse ligands more easily in the binding pocket once accommodated inside. On the other hand, SULT1A3, which shows more important substrate specificity, would need an additional molecular mechanism to ensure its specificity.

### 2.2. Clustering and Ensemble Docking

In order to obtain small representative conformational ensembles for SULT1A1 and SULT1A3, we performed conformational clustering based on the RMSD calculated on the heavy atoms of the binding pocket. Then, the clusters were ranked based on their size, and the most populous clusters were selected (see details in the Methods section). For SULT1A1+PAPS, the clustering was executed separately on the conformations generated by MD and MDeNM, resulting in 722 clusters in total and 94 clusters retained for MD, and 245 clusters in total and 87 clusters retained for MDeNM. In the case of SULT1A3, the clustering was carried out on the MD simulations of SULT1A3+PAPS+dopamine, using the same protocol, and finally retaining 104 MD clusters for ensemble docking. Our previous MD simulations of SULT1A3 without bound ligands have shown a large opening of the binding pocket due to the repulsing interaction between the two carboxylic groups of D86 and E146, which has not been appropriate for ensemble docking [10]. Hence, here, the clustering was performed on the MD-generated conformations of SULT1A3+PAPS+dopamine.

**Figure 5:**
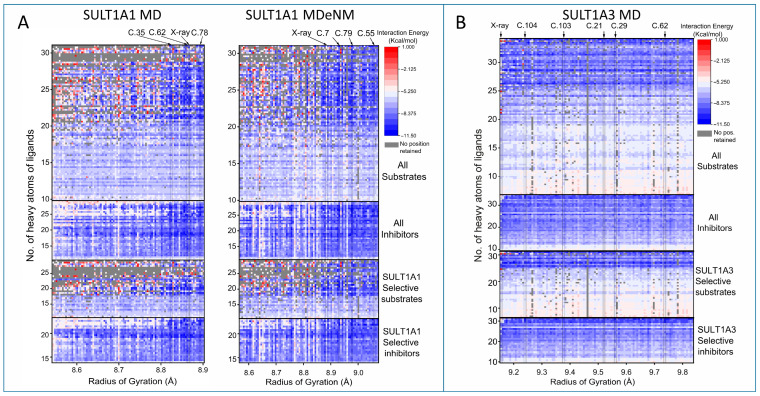
Docking score map of SULT1 substrates and inhibitors into protein cluster centroids. (**A**) Docking scores of SULT1A1on the MD and MDeNM centroids, ordered by the radius of gyration (RGYR) of the binding pocket (see residues in Supplementary Materials). (**B**) Docking scores for SULT1A3 on the MD centroids. Ligands in the ordinate axes are ordered by the number of their heavy atoms. Selective ligands indicate ligands not interacting with the other isoform. Blue to white colors indicate good predicted scores (for interaction energy), while red colors represent poor scores. Gray color indicates that docking has not produced a satisfactory pose of the substrate for the catalytic reaction. The results of the top 5 well-performing centroids and the X-ray conformation are marked with arrows.

The centroid conformations of the retained clusters were then used for the docking of substrates and inhibitors of the two enzymes. For SULT1A1, 131 previously collected substrates and inhibitors [10,26,27] were docked into the binding pocket of the centroids. For SULT1A3, we collected 143 substrates and inhibitors from several databases (see the Methods section for details). Figure 5 shows the heatmap of the docking results performed on the conformational ensembles of SULT1A1 and SULT1A3. The docking scores represent the interaction energy (IE) ranging from −11 kcal/mol (shown in blue) to +1 kcal/mol (shown in red). For the substrates, all docking scores were filtered; we employed a criterion of having the sulfate acceptor hydroxyl or primary amino functional group in the vicinity of the sulfate group of the co-factor PAPS within 5 Å. The best score satisfying the distance criterion was retained. In certain cases, indicated in gray, this filtering resulted in rejecting all the positions. The cluster centroids were ordered based on the expanse of the binding pocket, measured by the radius of gyration (RGYR) of the active site residues affecting the size of the pocket (the list of residues is given in the Supplementary Materials). For SULT1A1 (Figure 5A), the RGYR of the MD centroids ranges from 8.5 Å to 8.9 Å, and values over 8.8 Å correspond to a large opening of the binding pocket. For MDeNM, the RGYR ranges from 8.6 Å to 9.1 Å, which is in agreement with the wider conformational sampling. It can be seen that for larger ligands, SULT1A1 exhibits noticeably better IE for clusters, with RGYR values higher than 8.8 Å for both MD and MDeNM structures. Indeed, MDeNM achieved higher efficiency in generating more extended conformations, as discussed above.

The docking results for SULT1A3 are presented in Figure 5B. Interestingly, SULT1A3 shows considerably more open states of the binding pocket, ranging approximately from 9.1 to 9.9 Å. To note, the clustering for SULT1A3 was carried out on the MD trajectories in the presence of bound dopamine to hold the binding pocket appropriate for ligand docking. For comparison, the MD and MDeNM simulations of SULT1A1 were conducted without bound ligands. As expected, small ligands of SULT1A3 show worse IE than SULT1A1 due to the wider binding pockets generated. This confirms that the widely extended binding pocket is less efficient for accommodating small ligands. Being extremely flexible, it would be more difficult for SULT1A3 to establish strong interactions with small substrates.

Interestingly, for both isoforms, the inhibitors show better IE than the substrates. A reason for this might be that in the IEs calculated here, the reactivity of the substrates is not taken into account. In fact, the substrate reactivity is key for the catalytic reaction, and sometimes it makes a stronger contribution than the binding energy itself [28]. Similar trends can be observed when comparing selective substrates to selective inhibitors, namely inhibitors showing stronger IE than substrates. In addition, the selective substrates of SULT1A3 show a clear division of IE depending on their size.

To identify key residues interacting with the ligands, we have focused on protein conformations (marked in the top of Figure 5) that accommodate a large number of ligands with good interaction energies (indicated by a docking score lower than −5 kcal/mol). In the case of SULT1A1, three MD centroids and three MDeNM centroids were selected, docking 129 out of the 131 ligands with IE lower than −5 kcal/mol. For SULT1A3, five centroids were selected, docking 142 out of the 143 ligands with IE lower than −5 kcal/mol. Docking ligands on these centroids yielded better results than docking on the X-ray structures (for SULT1A1, 123 ligands were docked with IE lower than −5 kcal/mol, and 132 for SULT1A3) (see Figure S3). This can be attributed partially to the wider opening of the pocket, but is also a consequence of the relaxation of sidechains achieved with MD simulations.

### 2.3. Specific Interactions with Substrates

For the analysis of the interactions with particular residues in the binding pocket, we focused on the substrates, because their good positions can be selected considering the site of metabolism. Moreover, some inhibitors bind in allosteric sites, like catechins (naturally occurring flavonols) [29], or epigallocatechin gallate (EGCG) [30]. First, we analyzed the top-ranked competent docking poses of all selective substrates in the best centroids of SULT1A1 (the MDeNM centroid C7) and SULT1A3 (the MD centroid C103). In these two best protein centroids, and in the crystal structures (PDB ID 4GRA and 2A3R), the residues F24, F81, K106, H108, F142, H149, Y169, Y240, M248, and F255 are in contact with the docked selective substrates. Most of those are aromatic residues contributing to stacking the lipophilic substrates in the active sites of SULT1A1 and SULT1A3. The docking analyses also showed that the different residues interacting with the specific substrates are F76, F84, A146, and F247 for SULT1A1, and Y76, V84, D86, and E146 for SULT1A3. Obviously, these residues are important for the selectivity of the two SULT1 isoforms toward specific substrates.

**Figure 6:**
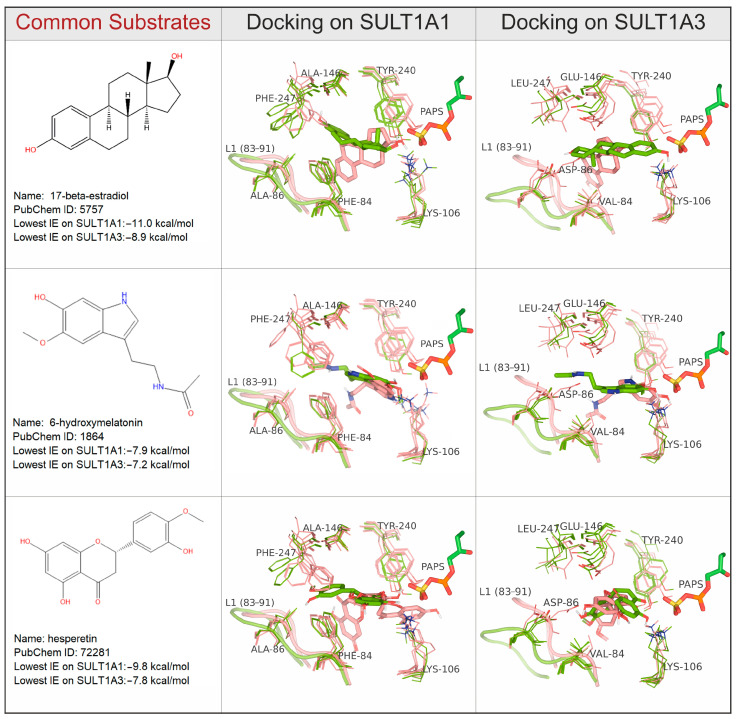
Key interactions of common substrates of SULT1A1 and SULT1A3. The selected best protein centroids noted in Figure 5 and the X-ray structures are shown. Favorable and unfavorable docking poses and the corresponding protein conformations are shown in green and salmon, respectively. Favorable docking poses are competent with the catalytic reaction. Highly flexible amino acid sidechains are depicted as lines, while the substrates and PAPS are depicted as sticks. Loop L1 is shown as a cartoon.

**Figure 7:**
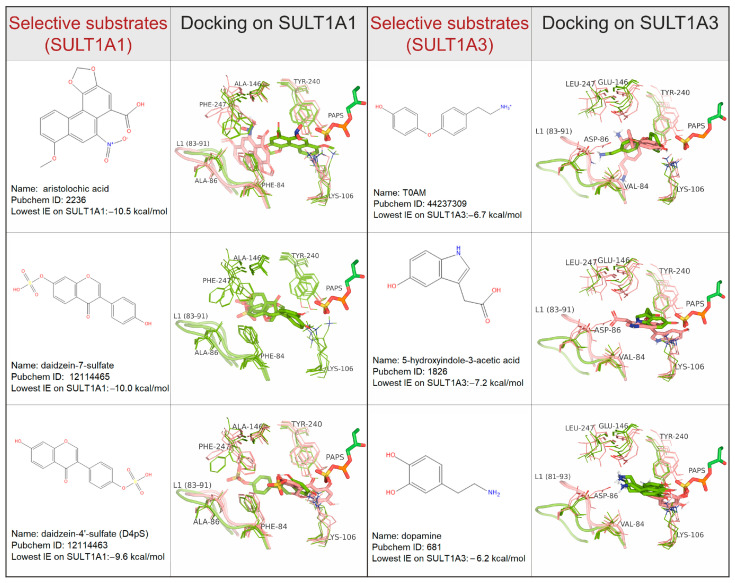
Key interactions of selective substrates of SULT1A1 and SULT1A3. The selected best protein centroids noted in Figure 5 and the X-ray structures are shown. Favorable and unfavorable docking poses and the corresponding protein conformations are shown in green and salmon, respectively. Favorable docking poses are competent within the catalytic reaction. Highly flexible amino acid sidechains are depicted as lines, while the substrates and PAPS are depicted as sticks. Loop L1 is shown as a cartoon.

Among the common substrates metabolized by both isoforms, structurally similar compounds were identified using ligand clustering (see Methods for details). This method resulted in 14 clusters containing polycyclic compounds (i.e., large substrates), and 6 clusters containing compounds with one cycle (i.e., small substrates). In Figure 6, we present three large common substrates, each representing a different cluster. An additional three large and three small common substrates are shown in the Supplementary Materials (Figures S4 and S5). Figure 7 shows three selective substrates of SULT1A1 and three selective substrates of SULT1A3 (chosen based on their diversity). To illustrate the most important interactions with the residues of the binding site, the substrates docked on the best protein cluster centroids (described previously and marked in Figure 5) are presented. The residues Y240 and K106, showing some flexibility, interact with the substrates in the two isoforms. It is seen that two aromatic residues, F247 and F84, systematically interact with all substrates via aromatic stacking in the case of SULT1A1. Although F247 has been previously suggested to participate in allosteric binding [31], our results indicate its importance also for the binding of substrates in the active site. The phenolic groups of F81 in SULT1A1 and F84 in SULT1A3 have been previously noted in the literature to be strong attractors of phenolic compounds, thus acting as a molecular clamp [21]. Interestingly, in our results, V84 of SULT1A3 shows some contacts only with the common substrates. Thus, we can conclude that residues 84, 106, and 240 are important for the interactions in the two isoforms, and thus contribute to their promiscuity.

Differently, SULT1A3 possesses two carboxylic groups of D86 and E146 that are well suited to stabilizing the binding of ligands containing positively charged groups, or containing hydrogen-bond donors, thus narrowing the range of accepted substrates. As expected, our modeling results clearly show that the ligand position is strongly dependent on the distance between the sidechains of D86 and E146. On the contrary, the small sidechains of A86 and A146 in SULT1A1 allow more diverse ligands to be accommodated in the active site. The substrate specificity of SULT1A3 has been explored in several site-directed mutagenesis studies, demonstrating that the variants E146A, D86A, and E89I of SULT1A3 exhibited the characteristics of SULT1A1, reducing Km for p-nitrophenol and increasing it for dopamine by two magnitudes [32,33].

## 3. Materials and Methods

### 3.1. Preparation of the Systems

The starting structures for both SULT1A1 and SULT1A3 were taken from Protein Data Bank [34] with IDs 4GRA [18] and 2A3R [35], respectively. The details of the system preparation of SULT1A1 were described in our previous article [23]. The structure of SULT1A3 contains PAP and dopamine, a selective substrate of this enzyme. Dopamine parametrization was determined using CGenFF (CHARMM General Force Field [36], ver: 2.2.0, Silcsbio, Baltimore, MD, US). For the SULT1A3 system preparation, we used the same protocol, including the replacement of the co-factor PAP by PAPS parametrized with CGenFF 2.2.0. PROPKA [37] (ver: 3.1, Jan H. Jensen Research Group, Copenhagen, Denmark) was used to determine the protonation states. All residues showed normal pK values, and the histidine residues of both isoenzymes showed single protonation at delta or epsilon nitrogen. Their protonation was assigned depending on their local environment. The online web tool CHARMM-GUI [38,39] was utilized to build a rectangular solvent box around the protein containing TIP3 water molecules, with dimensions of 82 Å × 82 Å × 82 Å, ensuring a minimal distance of 14 Å from the protein surface. NaCl concentration was set to 0.15 mol/L. Solvation details are given in Table S2 of the Supplementary Materials. Energy minimization was conducted with a series of progressively decreasing harmonic restraints applied to the atomic positions, beginning with steepest descent (SD), where the harmonic force constant decreased every 100 steps, with values of 50, 10, 1, and 0.1 kcal/mol/Å2. The harmonic restraints were then released, and three cycles of 250 steps of SD and adopted basis Newton–Raphson (ABNR) minimizations were performed, followed by a final cycle of 500 steps. CHARMM software [40] (ver: c44b2, Chemistry at HARvard Macromolecular Mechanics, Boston, MA, US) was used to perform the minimization, utilizing the additive all-atom CHARMM force field C36m [41]. The system was then heated and equilibrated in an NVT ensemble at 300 K for 100 ps, followed by a 5 ns NPT run at 1 atm pressure, using NAMD [42] (ver:2.13 Theoretical and Computational Biophysics Group, Urbana, IL, USA) and the same force field mentioned above. Langevin dynamics with a damping coefficient of 1 ps^−1^ was used for constant temperature control, while the Nose–Hoover method with a piston oscillation period of 50 fs; a piston oscillation decay time of 25 fs was used for constant pressure control. The integration time step was set to 2 fs. For the energy calculations, the dielectric constant was set to 1. Electrostatic interactions were calculated using the particle mesh Ewald (PME) method, with a grid spacing of 1 Å or less, having the order of 6. The real space summation was truncated at 12.0 Å, and the width of Gaussian distribution was set to 0.34 Å-1. Van der Waals interactions were reduced to zero using a ‘switch’ truncation operating between 10.0 and 12.0 Å. For SULT1A3, two systems were built: one with and one without dopamine. For the system not containing the dopamine, the substrate was simply removed from the X-ray structure.

### 3.2. MD Simulations and Analysis

All atom molecular dynamics simulations were performed for the systems SULT1A3+PAPS and SULT1A3+PAPS+dopamine using NAMD [42] with CHARMM force field C36m [41]. Three 500 ns long simulations were carried out starting from the equilibrated structures. For each run, random velocities were assigned according to the Maxwell–Boltzmann distribution at 300 K. The simulations were saved every 20 ps, resulting in 25,000 conformations per MD run, and a total of 75,000 conformations per system. The parameters for the 500 ns runs were identical to those used in the previously described 5 ns NPT equilibration. The details for the 200 ns long simulation of SULT1A1 are described in our previous work [23].

A principal component analysis (PCA) [43,44,45] was carried out on the simulations data using CHARMM [40]. All three runs were considered together in the PCA analysis for each system. After calculating the PCs, the structures of the simulation were projected onto the reduced space spanned by the first two PCs with the largest variances. Furthermore, the free energy landscapes (FEL) of the PCA projections were also calculated based on the 2D populational distributions. The most populated state was used as a reference for calculating free energy differences. The free energy difference (Δ*G_α_*) of a given state α was determined by considering the probability of the occurrence of the two states *P*(*q_α_*) and *P_max_*(*q*), given by the following equation:

$$\Delta G_{\alpha }=-k_{B}Tln\left[\frac{P\left(q_{\alpha }\right)}{P_{max}\left(q\right)}\right]$$

where *k_B_* is the Boltzmann constant, *T* is the temperature of the simulation, and *P*(*q_α_*) is an estimate of the probability density function obtained from the bi-dimensional histogram of the conformations. *P_max_*(*q*) is the probability of the most populated state.

### 3.3. Clustering

The quality threshold (QT) [46] algorithm was applied, as implemented in visual molecular dynamics (VMD) software [47] (ver:1.9.3 Theoretical and Computational Biophysics Group, Urbana, IL, USA). For SULT1A1, the clustering results of our previous study were used [23], which resulted in 94 MD and 86 MDeNM centroid conformations. For SULT1A3, MD simulations of SUL1A3+PAPS+dopamine were used for clustering, after the dopamine has been removed. Based on the RMSD difference calculated for the heavy atoms of the binding pocket (defined in the Supplementary Materials), the maximum cluster distance was set to 1.3 Å. The resulting clusters were ranked based on the number of conformations they contained, and the centroids of the first 104 clusters were selected for docking. These clusters covered 80% of all the frames from the three MD trajectories.

### 3.4. Ligand Collection and Preparation

Ligands of SULT1A1 had been previously collected [10]. A classification based on the ligands’ experimental activities was created, consisting of the following groups: substrates, inhibitors, selective substrates, and selective inhibitors. Ligand structures were converted to PDBQT format using Autodock Tools [48] (ver:4.2.6, Center for Computational Structural Biology, La Jolla, CA, USA), and Gasteiger charges were assigned. For SULT1A3, a new collection of ligands was conducted. The resulting chemical library consists of 143 substrates and inhibitors with biological activity (documented Km Ki or IC), taken from the following databases: PubChem (https://pubchem.ncbi.nlm.nih.gov/ accessed on: 13 April 2022), DrugBank (https://go.drugbank.com/ accessed on 13 April 2022), and Reaxys (https://www.reaxys.com/ accessed on 15 April 2022). Classification and ligand preparation followed the same protocol as for the ligands of SULT1A1.

### 3.5. Ensemble Docking

Docking experiments were performed with AutoDock Vina [49] (ver: 1.1.2, Center for Computational Structural Biology, La Jolla, CA, US). AutoDock Vina employs gradient-based conformational docking and an empirical scoring function predicting protein–ligand interaction energy (IE, in kcal/mol). The ensemble docking of SULT1A1 was performed in our previous study [23]. For SULT1A3, we performed docking simulations of the 104 MD centroid protein conformations. The protein conformations selected for docking were pre-processed using AutoDockTools [48], removing solvents, merging non-polar hydrogens, and assigning Gasteiger charges. The maximum number of binding modes was set to 20, and the exhaustiveness of the global search to 10. During the docking, the ligands were handled flexibly, while the protein and the co-factor were kept rigid. A grid box was centered in the binding pocket with dimensions of 24 Å × 24 Å × 24 Å, and the spacing was set to 1 Å. Filtering was performed to ensure that the distances between the substrate acceptor hydroxyl or primary amino functional group and the sulfate group of the co-factor PAPS and the catalytic residue H108 fell within 5 Å of all the substrates docked into SULT1A1 and SULT1A3.

### 3.6. Substrate Clustering

Substrates that are common to both isoenzymes were defined, and a diversity clustering was carried out, using FCFP_4 with a Tanimoto similarity criterion of 0.6, as implemented in Biovia Pipeline Pilot (ver: v.20.1, Dassault Systèmes, Vélizy-Villacoublay, France). Ligands containing a single aromatic ring and those with polycyclic or multiple-ringed structures were treated as different categories, called small and large ligands, respectively. A total of 14 clusters were obtained for large common substrates, and 6 for small common substrates.

## 4. Conclusions

SULT1A1 metabolized a broad range of substrates, and the more selective SULT1A3 showed distinctively different dynamics behavior. While SULT1A1 exhibits a relatively rigid structure by showing lower conformational flexibility except for the lip L1, the loops L2 and L3 of SULT1A3 are extremely flexible. One may speculate that the relative rigidity of SULT1A1 permeates to keep ligands in the binding pocket easily when accommodated inside, thus becoming more promiscuous than SULT1A3. Being more specific and highly flexible, the structure of SULT1A3 has particularities in the binding site, which are crucial for its substrate selectivity. Our ensemble docking successfully identified protein residues involved in the recognition of different substrates for the two isoforms. In the case of SULT1A1, F247 and F84 play an important role in the substrate accommodation and orientation, in particular for the large variety of small ligands. In the case of SULT1A3, our results confirmed the key role of the carboxylic groups of D86 and E146 in guiding the substrate specificity.

## Data Availability

All data of this study are available from the corresponding author upon reasonable request.

## Supplementary Materials

The following supporting information can be downloaded at: https://www.mdpi.com/article/10.3390/ijms242316900/s1.
