# Supplementary material for: Role of Conformational Dynamics of Sulfotransferases SULT1A1 and SULT1A3 in Substrate Specificity
Source: Int J Mol Sci. 2023 Nov 29;24(23):16900. doi: 10.3390/ijms242316900 (PMC10706399; doi:10.3390/ijms242316900)
Supplement: Supplementary file 1 [file ijms-24-16900-s001.zip › DToth_SULTs_Supporting_Information_proof.docx]

Supplementary Material

**Role of conformational dynamics of sulfotransferases SULT1A1 and SULT1A3 in substrate specificity**

D. Toth^1,2^, B. Dudas ^1,3^, M. A. Miteva^1*^, E. Balog^2*^

^1^CiTCoM UMR 8038 CNRS, INSERM U1268 MCTR, Université Paris Cité, Paris 75006, France

^2^Department of Biophysics and Radiation Biology, Semmelweis University, Budapest 1094, Hungary

^3^Department of Physics and Astronomy, University College London, London WC1E 6BT, United Kingdom

^*^ Correspondence:

[balog.erika@med.semmelweis-univ.hu](mailto:balog.erika@med.semmelweis-univ.hu) E.B; [maria.mitev@inserm.fr](mailto:maria.mitev@inserm.fr) M.A.M.

**SI Tables**

**Table S1.** **Variances along the first two Principal Components of the studied systems.**

|  | σ^2^(PC1) [Å^2^] | σ^2^(PC2) [Å^2^] |
| --- | --- | --- |
| SULT1A1+PAPS | 202.26 | 115.27 |
| SULT1A3+PAPS | 910.56 | 523.96 |
| SULT1A3+PAPS+dopamine | 665.10 | 268.37 |

**Table S2. Number of water molecules and ions in the studied systems.**

| **Solvent molecules/ions** | **SULT1A1+PAPS** | **SULT1A3+PAPS** | **SULT1A3+PAPS+dopamine** |
| --- | --- | --- | --- |
| Waters in the crystal structures | 39 | 53 | 53 |
| Waters in MD simulations | 15636 | 16284 | 16311 |
| Sodium ions in MD simulations | 50 | 56 | 54 |
| Chloride ion in MD simulations | 43 | 32 | 46 |

**SI Figures**


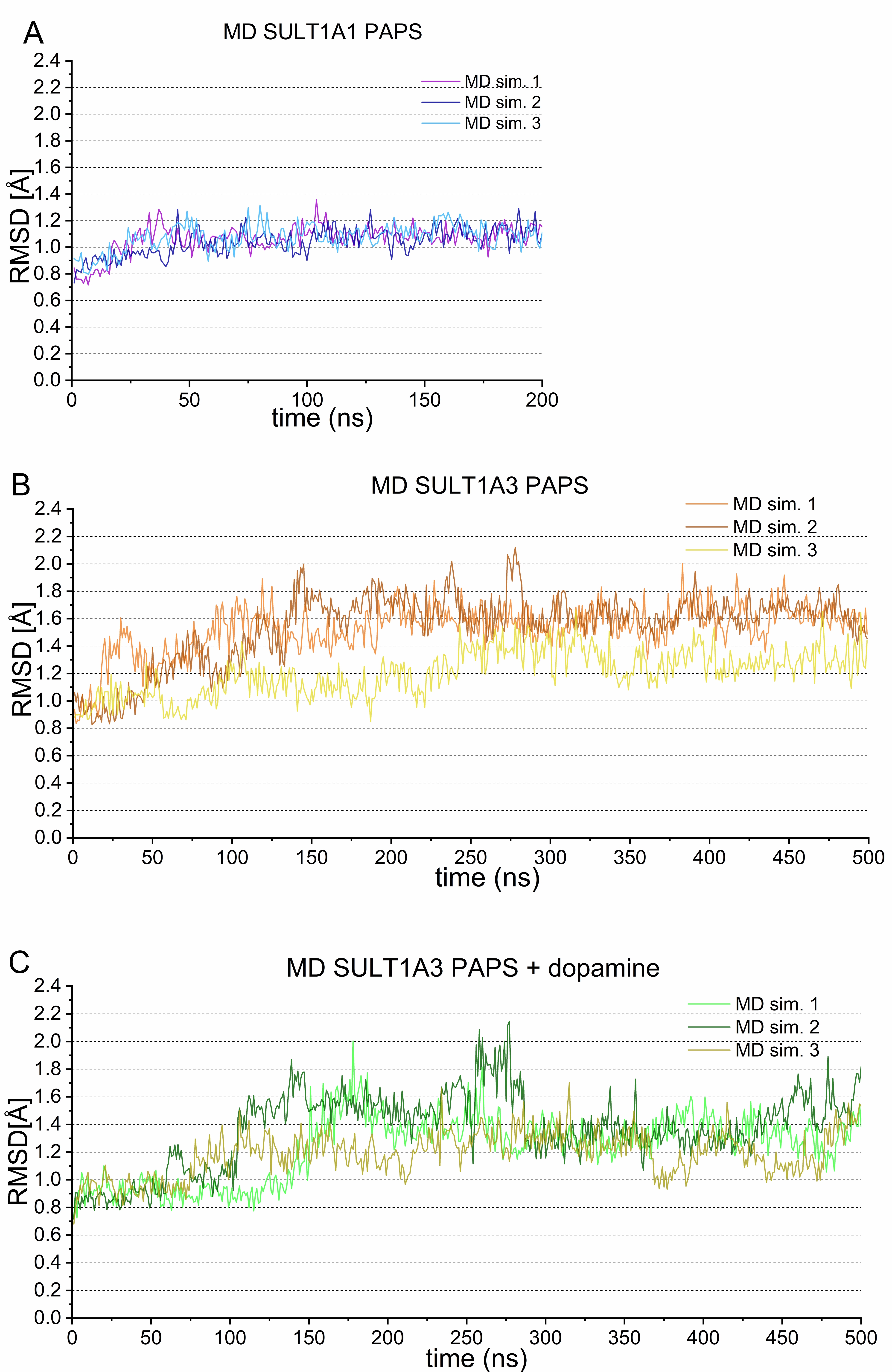


**Figure S1.** **The Root Mean Square Deviations (RMSD) as a function of time for SULT1A1+PAPS (A), SULT1A3+PAPS (B) and SULT1A3+PAPS+dopamine (C).** MD simulations were compared to the respective crystal structures and the RMSD was calculated for the backbone heavy atoms. For SULT1A1 200ns-long simulations, while for the two SULT1A3 systems, 500ns-long ns long simulations were carried out.


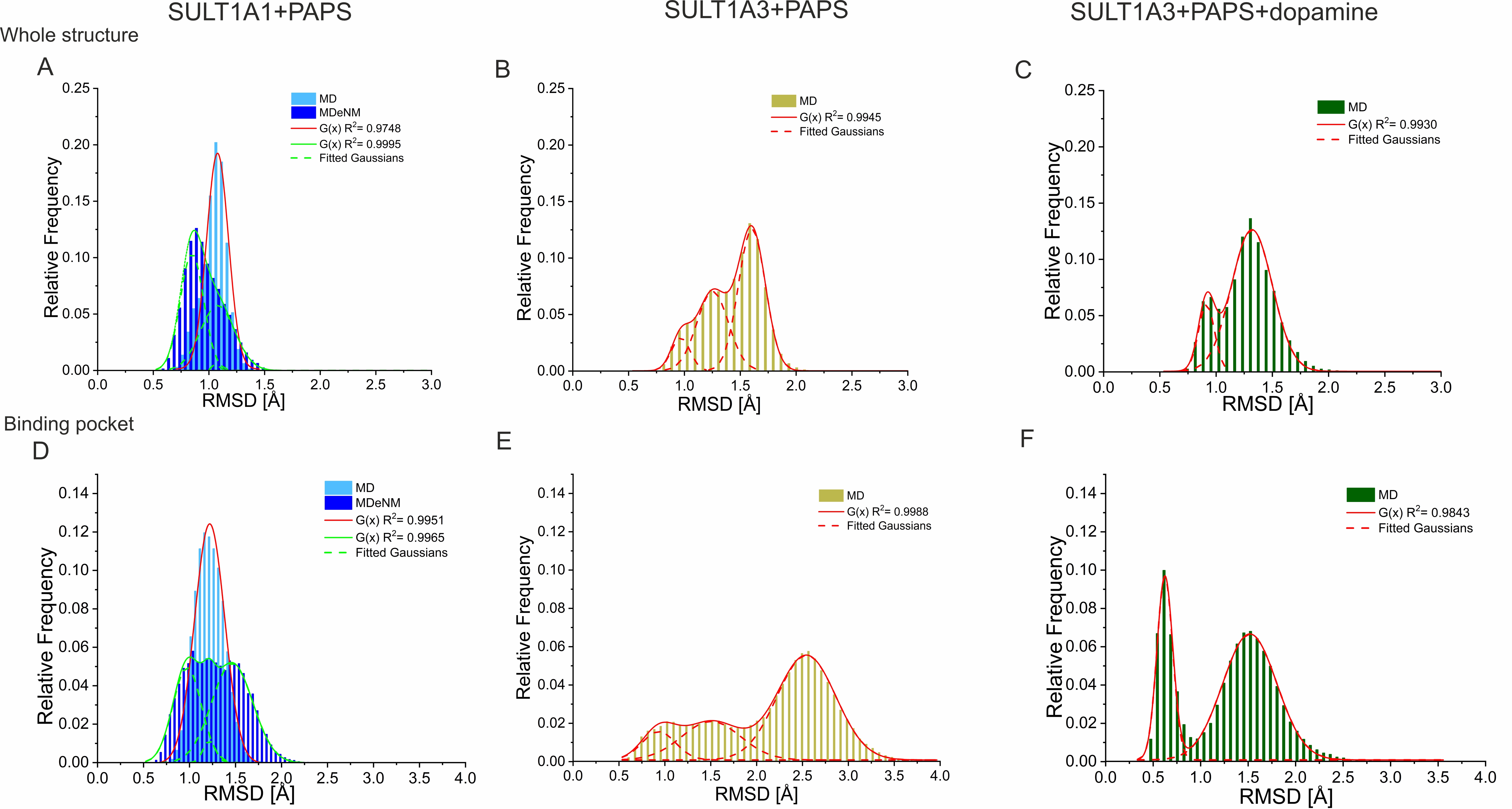


**Figure S2. Histogram of the Root Mean Square Deviations (RMSD) for the SULT1A1 and SULT1A3 simulations.** Gaussian fits on the data are shown by dashed lines, while the sum of the Gaussians is shown by continuous lines. The R^2^ of the fits are also indicated.


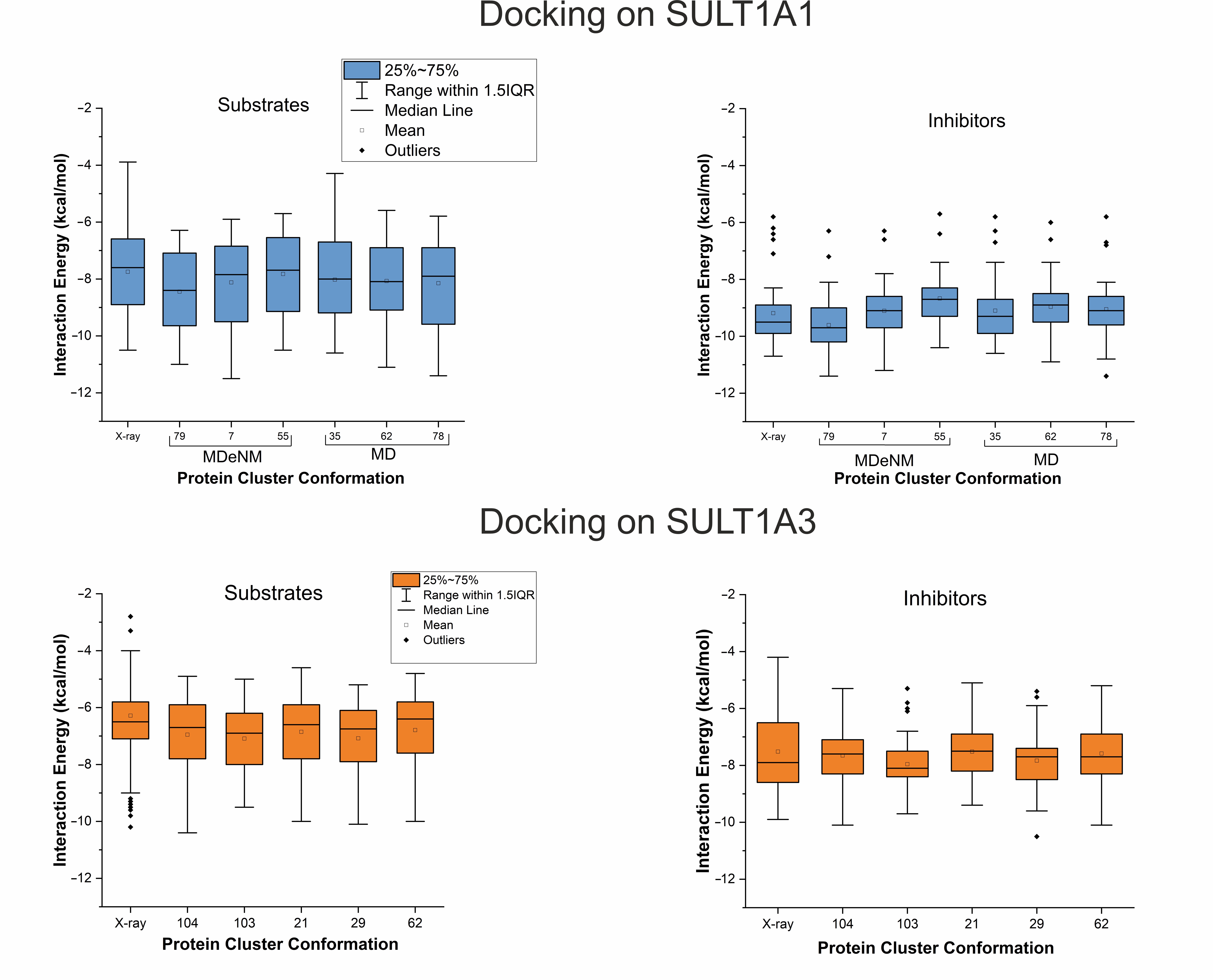


**Figure S3.** **Docking scores (Interaction Energy) for SULT1A1 (in blue) and SULT1A3 (in orange) on the cluster centroids showing best docking results.** The first box in each diagram shows the results of docking performed on the x-ray structure.


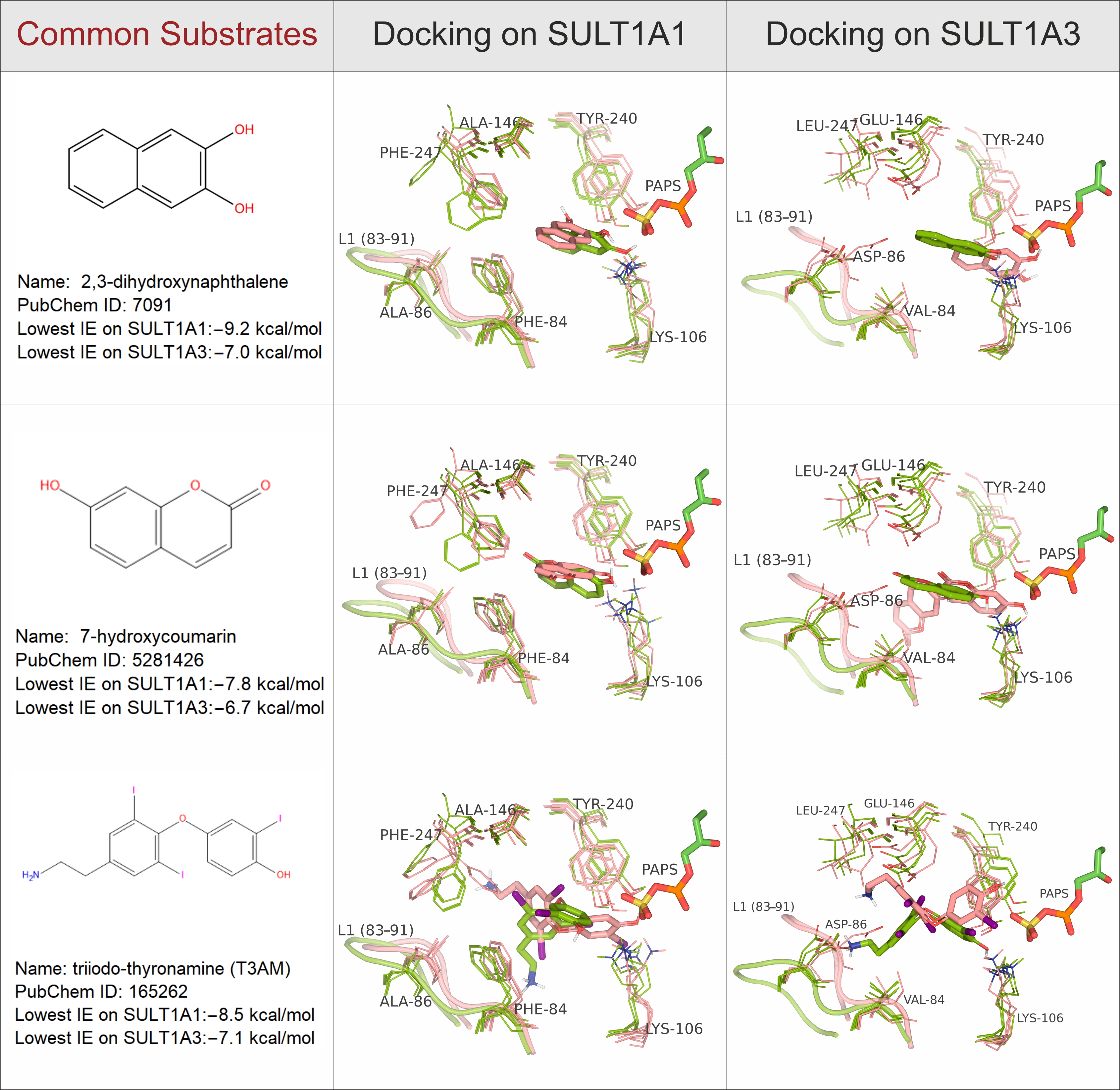


**Figure S4.** **Key interactions of large common substrates of SULT1A1 and SULT1A3.** Additional polycyclic compounds, i.e. large common substrates are presented here, which are sulfonated by both SULT1A1 and SULT1A3. The selected best protein centroids noted in Fig.4 and the X-ray structures are shown. Favorable and unfavorable docking poses and the corresponding protein conformations are shown in green and salmon, respectively. Favorable docking poses are competent with the catalytic reaction. Highly flexible amino acid sidechains are depicted as lines, while the substrates and PAPS are depicted as sticks. Loop L1 is shown as cartoon.


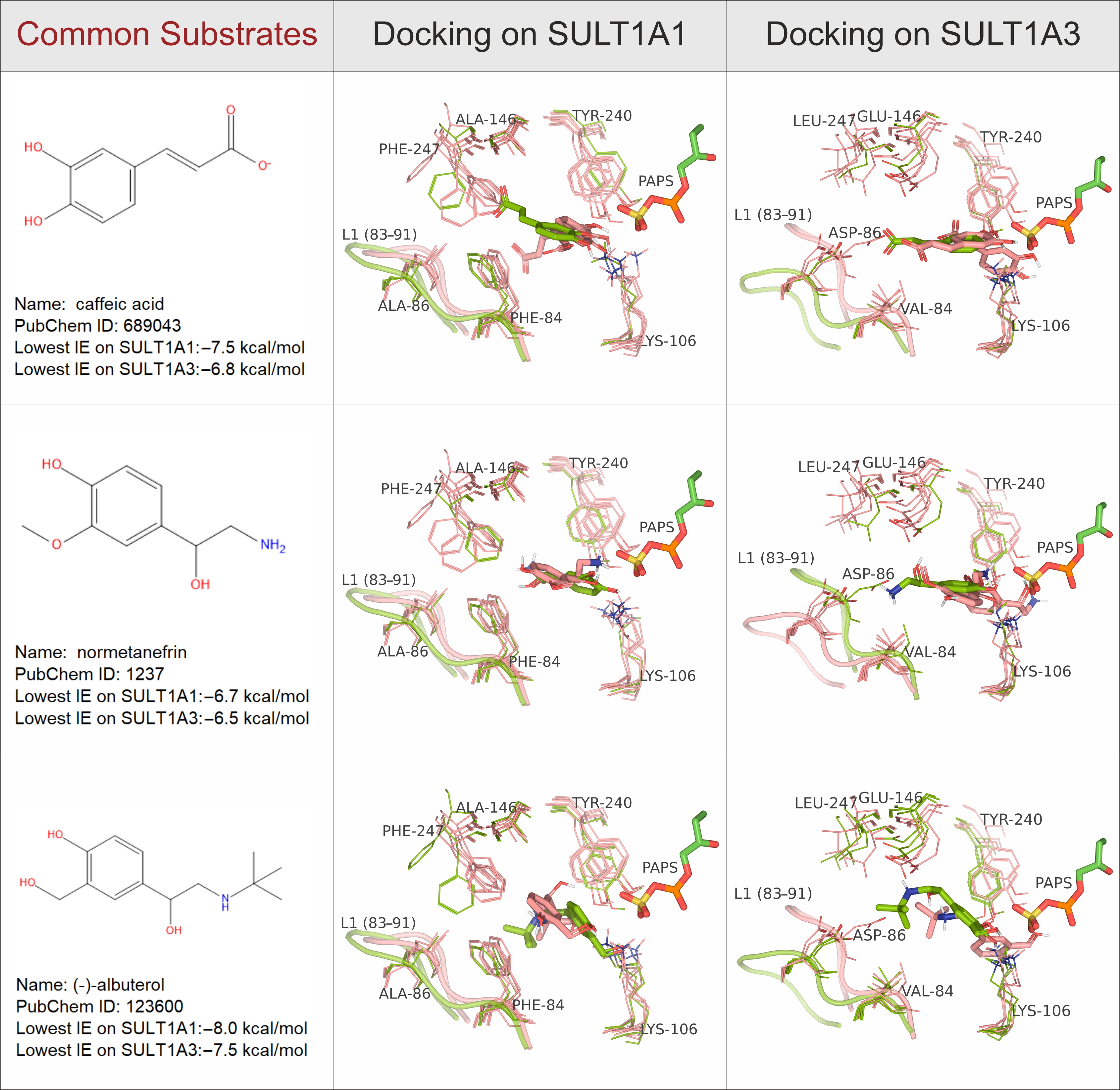


**Figure S5**. **Key interactions of small common substrates of SULT1A1 and SULT1A3.** Additional compounds containing a single aromatic ring, i.e. small common substrates are presented here, which are sulfonated by both SULT1A1 and SULT1A3. The selected best protein centroids noted in Fig.4 and the X-ray structures are shown. Favorable and unfavorable docking poses and the corresponding protein conformations are shown in green and salmon, respectively. Favorable docking poses are competent with the catalytic reaction. Highly flexible amino acid sidechains are depicted as lines, while the substrates and PAPS are depicted as sticks. Loop L1 is shown as cartoon.

**SI Lists**

**SULT1A1:**

**List of residues forming the binding pocket:**

I21, F24, T45, Y46, P47, F81, F84, K85, A86, I89, K106, T107, H108, F142, A146, K147, V148, H149, Y169, Y240, T241, T242, V243, P244, Q245, E246, F247, M248, D249, H250, F255

**List of residues used to calculate RGYR of the binding pocket:**

I21, F24, P47, K48, F76, F81, F84, P87, I89, K106, H108, Y139, F142, A146, V148, H149, S168, Y169, Y240, F247, M248, F255

**SULT1A3:**

**List of residues forming the binding pocket:**

I21, Y23, F24, T45, Y46, P47, F76, F81, V84, N85, D86, E89, K106, S107, H108, F142, E146, K147, A148, H149, Y169, Y240, T241, T242, V243, P244, Q245, E246, L247, M248, D249, H250, F255,

**List of residues used to calculate RGYR of the binding pocket:**

I21, F24, P47, K48, F76, F81, V84, D86, P87, E89, K106, H108, Y139, F142, E146, A148, H149, S168, Y169, Y240, L247, M248, F255

**SI Movies**

**Supplementary Movie 1.** MD simulation trajectories (Sim.1, Sim.2, Sim.3) of SULT1A1+PAPS. The loops L1, L2 and L3 are colored in orange, green and magenta, respectively.

**Supplementary Movie 2.** MD simulation trajectories (Sim.1, Sim.2, Sim.3) of SULT1A3+PAPS. The loops L1, L2 and L3 are colored in orange, green and magenta, respectively.

**Supplementary Movie 3.** MD simulation trajectories (Sim.1, Sim.2, Sim.3) of SULT1A1+PAPS+dopamine. The loops L1, L2 and L3 are colored in orange, green and magenta, respectively.
